# Non-Destructive Diagnostics of Concrete Beams Strengthened with Steel Plates Using Modal Analysis and Wavelet Transform

**DOI:** 10.3390/ma14113014

**Published:** 2021-06-02

**Authors:** Magdalena Knak, Erwin Wojtczak, Magdalena Rucka

**Affiliations:** 1 Department of Mechanics of Materials and Structures, Faculty of Civil and Environmental Engineering, Gdańsk University of Technology, Narutowicza 11/12, 80-233 Gdańsk, Poland; s168197@student.pg.edu.pl (M.K.); erwin.wojtczak@pg.edu.pl (E.W.); 2 EkoTech Center, Gdańsk University of Technology, Narutowicza 11/12, 80-233 Gdańsk, Poland

**Keywords:** non-destructive testing, damage detection, vibrations, modal analysis, continuous wavelet transform, concrete beam, strengthening, adhesive joint, debonding

## Abstract

Externally bonded reinforcements are commonly and widely used in civil engineering objects made of concrete to increase the structure load capacity or to minimize the negative effects of long-term operation and possible defects. The quality of adhesive bonding between a strengthened structure and steel or composite elements is essential for effective reinforcement; therefore, there is a need for non-destructive diagnostics of adhesive joints. The aim of this paper is the detection of debonding defects in adhesive joints between concrete beams and steel plates using the modal analysis approach. The inspection was based on modal shapes and their further processing with the use of continuous wavelet transform (CWT) for precise debonding localization and imaging. The influence of the number of wavelet vanishing moments and the mode shape interpolation on damage imaging maps was studied. The results showed that the integrated modal analysis and wavelet transform could be successfully applied to determine the exact shape and position of the debonding in the adhesive joints of composite beams.

## 1. Introduction

A significant part of building objects is made of concrete, which is continuously degraded as a result of environmental and loading conditions as well as natural ageing of the material. Therefore, in many situations, structural reinforcements are used to minimize the negative effects of long-term operation and developing damage. For this purpose, various systems can be used, among which the use of externally bonded reinforcement (EBR) is one of the most effective [1,2,3,4]. EBRs usually have a form of adhesively bonded elements made of steel (e.g., plates, rods, flat bars) or composites like fiber-reinforced polymers (e.g., tapes or mats). In such connections, it is crucial to provide an adequate quality of the bonding as well as further assessment of its condition and monitoring. Recently, various non-destructive testing (NDT) and structural health monitoring (SHM) approaches have been increasingly and widely incorporated to improve the safety of structures by precise damage detection, identification, and visualization [5,6]. In the existing literature, there are many examples of successful application of non-invasive techniques for diagnostics of adhesive joints [7], especially utilizing thermography [8,9,10,11], reflectometry [12] or ultrasonic waves [13,14,15,16,17].

In this study, an evaluation of a steel–concrete composite beam was presented. Particular attention was paid to the visualization of defects between a reinforcing plate and a concrete beam. The research was carried out using vibrations and their further processing. Vibration-based methods belong to the most popular and widely used damage detection techniques for decades [18,19,20,21] and they are still intensively developed [22]. Many researchers use modal analysis as a diagnostic tool (e.g., [23,24,25,26,27,28,29,30]). Changes in modal parameters, such as natural frequencies, mode shapes, or damping coefficients, make it possible to monitor the condition of structures. Most often, the results obtained from mode shapes are enhanced by the calculation of modal curvatures [25,26,30]. To increase the efficiency of damage detection and localization, more and more researchers decide to use wavelet analysis [26,27,28,29,30,31,32,33,34,35,36,37,38,39]. Wavelet-based methods allow precise localizing and imaging defects, which is not always possible directly through modal analysis. In previous works, wavelet analysis has been applied for the detection of different kinds of defects in various structures, such as single or multiple notches in beams [25,26,29,30,31,32,33,34,35,36], spatial defects (in the form of local reduction of thickness) in plates [24,33,37,39] or impact damages in plates [38,39]. However, the literature on the non-invasive diagnostics of adhesive joints using vibration methods is limited and the problem of damage imaging in such joints by wavelet analysis has not been thoroughly considered. Recently, Yang and Oyadiji [28] used modal analysis and discrete wavelet transform of modal frequency curves to identify debonding in adhesive joints in two-layer bonded aluminum beam samples. To the best of the authors’ knowledge, there is no research on integrated vibration and wavelet-based damage detection in steel–concrete adhesive connections.

This study presents a vibration-based condition assessment of the adhesive connection between a concrete beam and a steel plate. Experimental and numerical investigations were performed on a beam with a perfectly bonded joint as well as three beams with debonding defects of different areas. The diagnostic procedure used mode shapes and their further processing based on continuous wavelet transform for precise debonding localization and imaging. The influence of the number of wavelet vanishing moments and the mode shape interpolation on damage imaging maps was studied.

## 2. Materials and Methods

### 2.1. Object of Research

**Figure 1:**
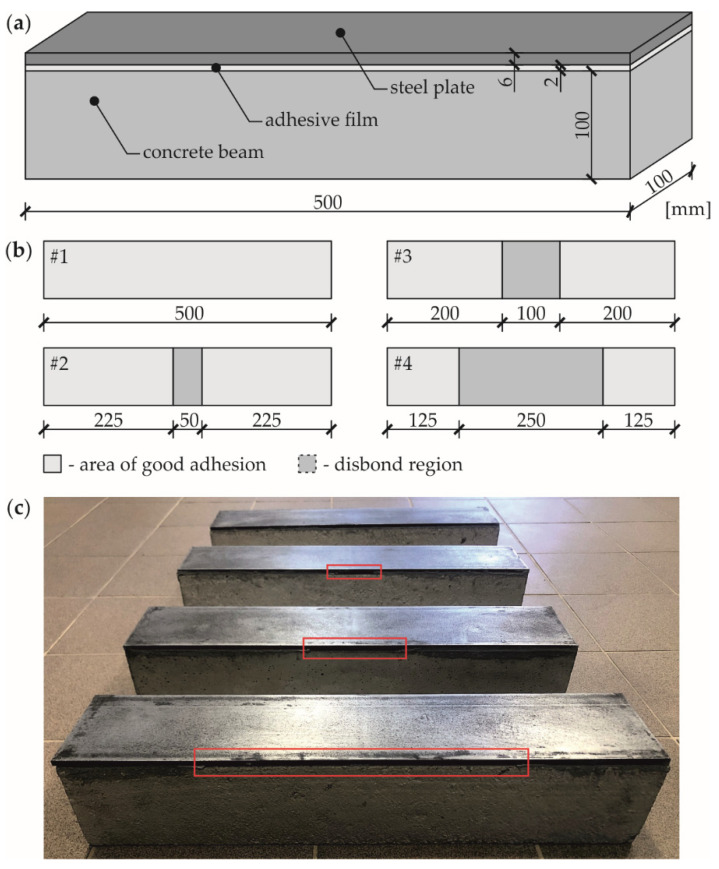
Object of research: (**a**) Specimen geometry; (**b**) variants of defects; (**c**) photograph of experimental samples.

The object of research was a multilayer sample (Figure 1a) consisting of a concrete beam (class C30/37) with a square cross-section of 100 *×* 100 mm^2^, and a length of 5000 mm, an adhesive film with a thickness of 2 mm and a steel plate with dimensions of 6 *×* 100 *×* 5000 mm^3^. The material characteristics are given in Table 1. Four specimens (Figure 1b) were prepared: An intact composite beam (with no damage, #1) and three beams (#2-4) with the increasing percentage of debonding, 10%, 20% and 50%, consecutively. Each defect was arranged as a lack of an adhesive film by sticking a Teflon (PTFE) tape to the appropriate area of the joint. Before preparation of each sample, the contacting surfaces of the concrete beam and steel plate were accurately cleaned using Loctite-7063 cleaner (Henkel, Dusseldorf, Germany). Immediately after that, both elements were joined using Sikadur 30 Normal adhesive (Sika, Baar, Switzerland). The surface of the beam was primed with glue and then the adhesive layer was applied in a domed shape. This allowed the elimination of any possible air voids from the joints during attaching the plate to the concrete beam. The prepared samples are presented in Figure 1c.

### 2.2. Experimental Procedure

**Figure 2:**
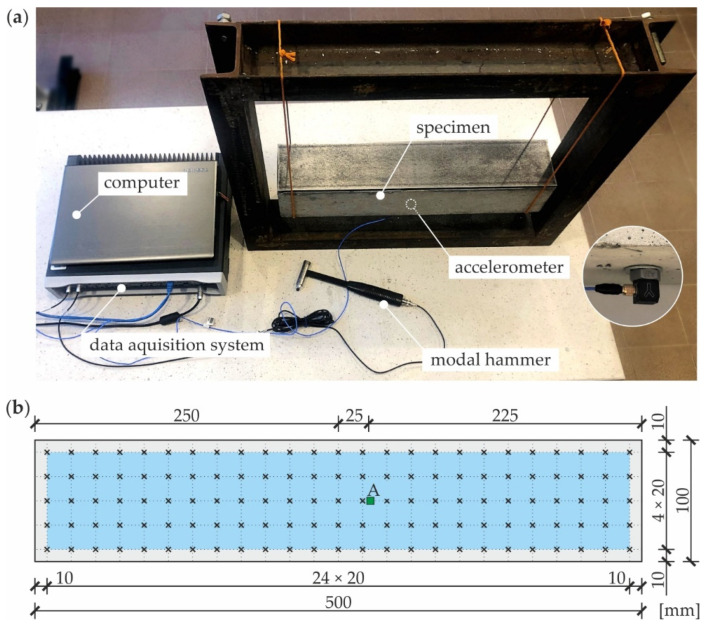
Experimental setup (**a**) and scheme of measurement grid (**b**).

Dynamic parameters of the tested specimens (i.e., natural frequencies and modal shapes) were determined using the experimental modal analysis (EMA) approach, in which both excitation and response signals were measured. In the study, an impact test was conducted with the use of a modal hammer. The experimental setup for EMA is shown in Figure 2a. The specimen was suspended from both sides on elastic strings to simulate free boundary conditions. Piezoelectric accelerometer 356A15 (PCB Piezotronics, Inc., Depew, NY, USA) was used for the measurement of vibrations. The properties of the sensor used are as follows: sensitivity 10.2 mV/(m/s^2^), measurement range ±490 m/s^2^, resonant frequency ≥25 kHz and frequency range 2–5000 Hz. The accelerometer was attached to the bottom surface of the beam at point A located 25 mm from the center. The dynamic pulse load was induced by the modal hammer 086C03 (PCB Piezotronics, Inc., Depew, NY, USA) with the following parameters: sensitivity 2.25 mV/N, measurement range ±2224 N, resonant frequency ≥22 kHz. A medium tip was applied with the hammer enabling the excitation of vibrations within the frequency range up to approximately 2800 Hz. A single measurement was performed at each of 125 points. The points of impact, marked in Figure 1b, were distributed on the top surface of the specimen in a regular square grid having 5 rows and 25 columns, resulting with a resolution of 20 mm in both directions. Data acquisition and signal conditioning were performed by LMS SCADAS portable system (Siemens, Leuven, Belgium). Natural frequencies and modal shapes were determined based on the frequency response function (the accelerance in this case) given as the ratio of an output acceleration signal to an input force signal [33,40,41]. The estimation of modal parameters was performed using a peak picking method. Mode shapes were determined by measuring the peak amplitude of the imaginary part of the frequency response function.

### 2.3. Numerical Modelling

**Figure 3:**
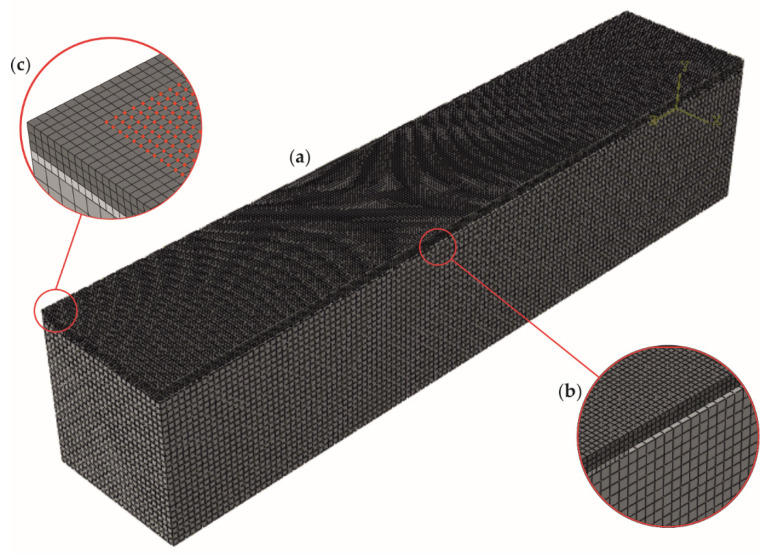
Numerical model (**a**) with a close-up of damage (**b**) and measurement grid (**c**) based on the #3 model.

The numerical calculations were conducted with the use of the finite element method (FEM) in Abaqus software. Modal analysis was performed on the three-dimensional numerical models (Figure 3a) prepared based on the geometry and materials of the physical samples. Material parameters (Table 1) were used to apply a linearly elastic, isotropic, homogeneous material model to all structural elements (steel plate, adhesive film, and concrete beam), all being independent parts. The rigid surface-to-surface *tie* connection was used for bonding the contacting regions of each part. Three-dimensional eight-node linear brick finite elements with reduced integration (C3D8R) were used to mesh all parts. The mesh grid has a size of 2 *×* 2 *×* 2 mm^3^ (for steel plate and adhesive film) and 4 *×* 4 *×* 4 mm^3^ (for the concrete beam). The debonding in models #2–4 was modelled as a gap in the adhesive film (see Figure 3b), which relates to the lack of glue in the physical samples. The frequency procedure (linear perturbation theory) was performed to determine the natural frequencies and the corresponding mode shapes. The results (normalized displacements) were read from a regularly gridded square mesh with a global size of 2 mm located on the upper surface of a steel plate, covering a central area of 80 × 480 mm^2^ with a margin of 10 mm at all edges (see Figure 3c). The additional coarser mesh was assumed with the size of 20 mm to coincide with the experimental measurements (cf. Figure 2b).

### 2.4. Continuous Wavelet Transform for Mode Shape Processing

The continuous wavelet transform (CWT) of a given signal *f*(*x*) is the inner product of the signal function with the shifted and scaled wavelet function [42]. It can be calculated with respect to the formula:

(1) $$Wf(u,s)=\langle f,\psi_{u,s}\rangle =\frac{1}{\sqrt{s}}\int_{-\infty }^{+\infty }f(x)\;\psi^{*}\left(\frac{x-u}{s}\right)dx,$$

where *x* is the distance variable, the parameters *s* and *u* are scale and translation, respectively and ψ^*^(x) is the complex conjugate of the wavelet function, which is required to have zero average:

(2) $$\int_{-\infty }^{+\infty }\psi (x)dx=0.$$

For specific values of *s* and *u*, *Wf* (*u*,*s*) is called the wavelet coefficient for the wavelet function *ψ_u,s_(x*).

An important property of wavelets is their ability to react to any discontinuities comprised in a signal. For the detection of signal singularities, the so-called vanishing moments are crucial. A particular wavelet having *n* vanishing moments is characterized by the orthogonality to polynomials up to degree *n*–1:

(3) $$\int_{-\infty }^{+\infty }x^{k}\psi \left(x\right)dx=0,\overset{}{\underset{}{}}k=0,1,2,\ldots ,n-1.$$

It can be proved that a wavelet with *n* vanishing moments can be rewritten as the *n*-th order derivative of a function [42]:

(4) $$\psi (x)=\frac{d^{n}\theta (x)}{dx^{n}}.$$

As a consequence, the wavelet transform given by Equation (1) can be expressed as a multiscale differential operator:

(5) $$Wf(u,s)=s^{n}\frac{d^{n}}{du^{n}}(f*\bar{\theta_{s}})(u),$$

where the notation $\left(f*\bar{\theta_{s}}\right)$ denotes the convolution of functions *f* and $\bar{\theta_{s}}$. Therefore, the wavelet transform is the *n*-th derivative of the signal smoothed by the function $\bar{\theta_{s}}(x)$ at scale *s*:

(6) $$\bar{\theta_{s}}(x)=\frac{1}{\sqrt{s}}\theta \left(\frac{-x}{s}\right).$$

If a signal has a singularity at a certain point, then the wavelet coefficients have relatively large values. Singularities are detected at coordinates where the CWT modulus maxima converge at fine scales [42]. When the scale is large, the only detection of large variables is possible, because the convolution with $\bar{\theta_{s}}(x)$ removes small signal fluctuations. On the other hand, when the scale decreases, the wavelet coefficients may have no maxima in the vicinity of the singularity [42]. Therefore, the proper selection of the scale is crucial.

Several families of wavelets are described in the literature; in this study, wavelets from the Gaussian wavelet family were used due to their high efficiency in the detection of singularities [31,35]. The family of Gaussian wavelets is based on the Gaussian function $g(x)=C_{a}e^{-x^{2}}$, by taking the *a*-th derivative of *g*(*x*) [43]. The first four wavelets from the Gaussian family have the following form [31]:

(7) $$\psi (x)={\left(-1\right)}^{1}2\sqrt[{4}]{2/\pi }xe^{-x^{2}}$$

(8) $$\psi (x)={\left(-1\right)}^{2}\frac{2\sqrt[{4}]{2/\pi }}{\sqrt{3}}(1-2x^{2})e^{-x^{2}}$$

(9) $$\psi (x)={\left(-1\right)}^{3}\frac{-4\sqrt[{4}]{2/\pi }}{\sqrt{15}}(3x-2x^{3})e^{-x^{2}}$$

(10) $$\psi (x)={\left(-1\right)}^{4}\frac{4\sqrt[{4}]{2/\pi }}{\sqrt{105}}(3-2x^{2}+4x^{4})e^{-x^{2}}$$

Equations (7)–(10) describe wavelets *gaus1*, *gaus2*, *gaus3*, and *gaus4* having 1, 2, 3 and 4 vanishing moments, respectively. *Gaus1* wavelet enables to extract information of the first-order derivative of *f*(*x*), *gaus2* represents the curvature of the function, while *gaus3* and *gaus4* correspond to higher-order derivatives. These properties of wavelets will be used in damage detection in the following section.

## 3. Results and Discussion

### 3.1. Modal Analysis—Natural Mode Shapes

**Figure 4:**
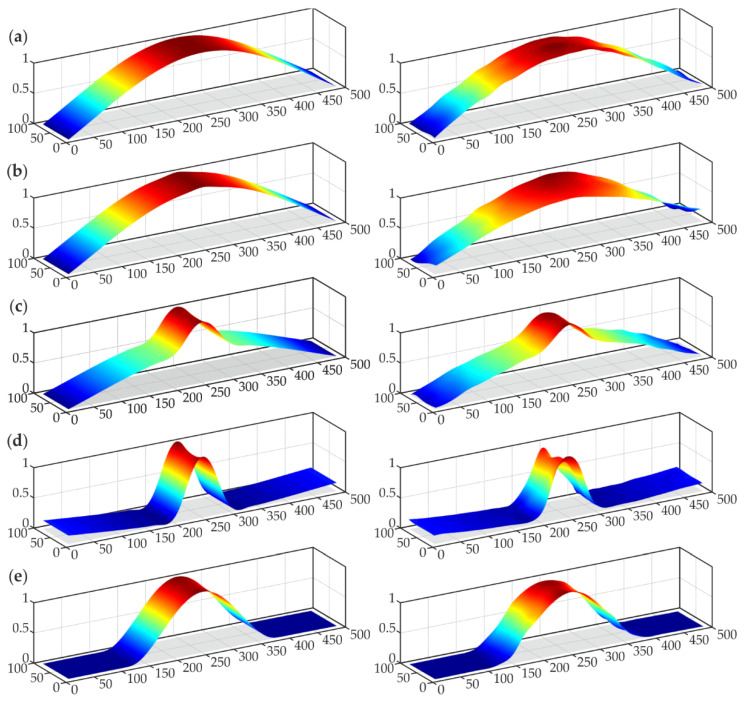
Normalized numerical (left column) and experimental (right column) mode shapes (dimensions in [mm]): (**a**) sample #1, mode 1; (**b**) sample #2, mode 1; (**c**) sample #3, mode 1; (**d**) sample #3, mode 2; (**e**) sample #4, mode 1.

The natural modes characterized by eigenfrequencies in the range of 0–2500 Hz were determined for all samples (based on the experimental results). Within this frequency range, five modes were taken into consideration, one for each beam (with the exception being sample #3, for which two modes were determined). Because the signals were measured only perpendicularly to the beam surface, all modes were related to the flexural deformations. Based on the obtained eigenfrequencies and mode shapes, the corresponding numerical modes were matched. The comparison of numerical and experimental frequencies is presented in Table 2, whereas the mode shapes are shown in Figure 4. The consistency of both approaches was evident. The differences between eigenfrequencies were below 10%, which allows stating that the experiments were conducted correctly, and also the numerical calculations were performed properly. Additionally, the modal assurance criterion (MAC) was applied to evaluate the degree of consistency between numerical and experimental mode shapes. Obtained MAC values ranged from 0.9782 to 0.9991, indicating very good agreement.

It can be observed that the value of the first natural frequency slightly decreased with the size of debonding between beams #1–3. For sample #4, the decrease became dramatic, because the area of the defect covered 50% of the whole joint, thus the steel plate could oscillate as an independent part. It is not surprising that there were no disturbances in the first mode shape for beam #1 without damage (Figure 4a). For sample #2 (Figure 4b), the defect was barely visible in the numerical mode, whereas the experimental one did not reveal any disruption, thus its exact size could not be assessed. On the other hand, both modes of beam #3 (Figure 4c,d) gave useful information about the presence and approximate size of the damage. The deformation in the area of debonding was greater than in the good adhesion part of the joint. It has to be noted that mode 1 corresponds to the global character of vibrations, the displacements are comparable in the area of good adhesion and debonding. Nevertheless, mode 2 has the local character, because vibrations in the debonding region are significantly higher than in the adjacent part of the specimen. The first mode shape of beam #4 (Figure 4e) also revealed the presence of the defect. What is interesting, no significant deformation was detected in the area of the properly prepared joint while comparing with the damaged part. This effect was not observed for the previous samples, where the oscillations had a global character. For beam #4, it can be stated that the first mode corresponded to the independent oscillation of the steel plate, thus the oscillations could be considered local. This difference stayed in agreement with the fact of a clear difference in the first eigenfrequency between beam #4 and the remaining ones. Summarizing, the analysis of mode shapes could provide an initial assessment of damage presence, especially in the case of large damage; however, further data processing is required to better visualize the defects.

### 3.2. Wavelet Transform-Based Damage Imaging

**Figure 5:**
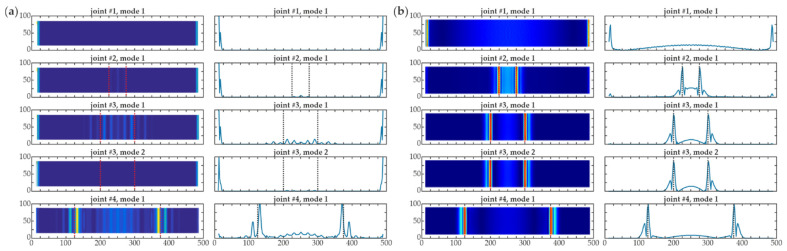
Influence of boundary effect to damage imaging (numerical results mesh 2 mm, no additional interpolation) for all joints #1–#4 (dimensions in [mm])—map and central cross section: (**a**) continuous wavelet transform (CWT) without extrapolation, wavelet *gaus4*, *s* = 2; (**b**) CWT with extrapolation, wavelet *gaus4*, *s* = 2.

Continuous wavelet transform was used to identify the actual shape and position of debonding areas in the adhesive joints. The prepared maps presenting mode shapes were separated into five vectors situated along the length of the specimen (cf. Figure 2b), creating five single lines. For each line, the calculations of the CWT were conducted independently, using a program written in MATLAB^®^ environment [44]. Firstly, to avoid boundary effects, extrapolation was applied. Figure 5 shows the efficiency of extrapolation in the elimination of the potential edge effects. In raw data (Figure 5a, no extrapolation) the intensification of CWT values is observed near the edges, thus the damage identification becomes problematic. This is because edge values are relatively high compared to the ones indicating the presence of defects. The performed extrapolation allowed eliminating this effect, resulting in sufficient damage imaging. It is also worth noting that edge effects are more visible for smaller defects.

**Figure 6:**
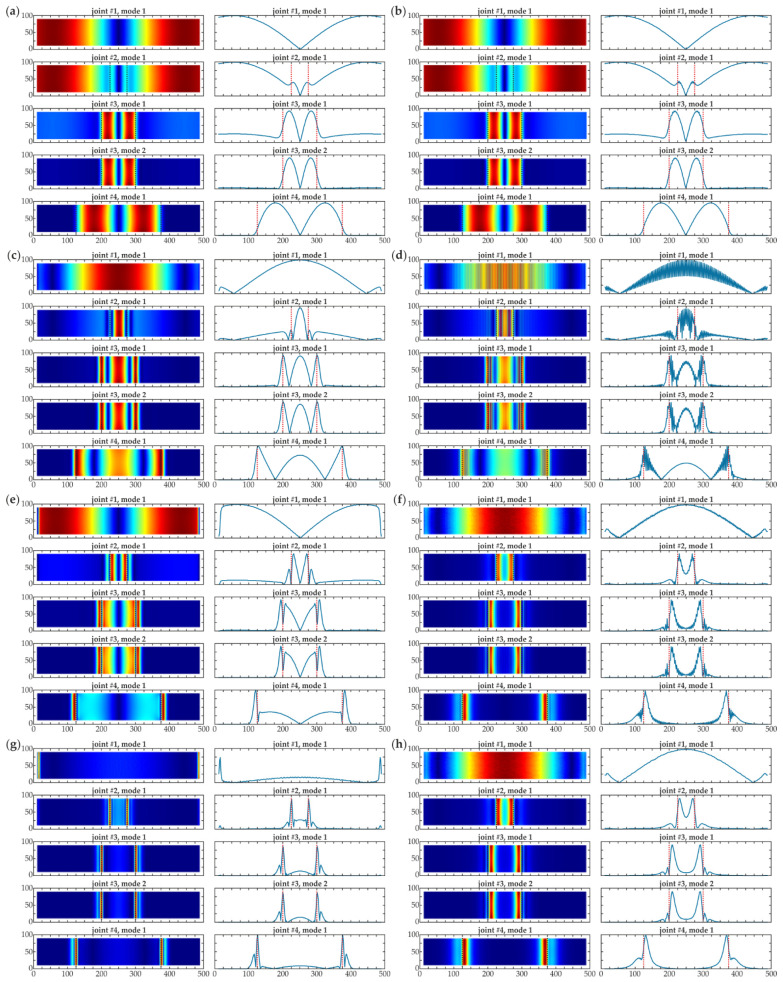
Damage imaging for numerical results (mesh 2 mm, no additional interpolation) for all joints #1–#4 (dimensions in [mm])—map and central cross section: (**a**) CWT, wavelet *gaus1*, *s* = 2; (**b**) first derivative; (**c**) CWT, wavelet *gaus2*, *s* = 2; (**d**) second derivative; (**e**) CWT, wavelet *gaus3*, *s* = 2; (**f**) third derivative; (**g**) CWT, wavelet *gaus4*, *s* = 2; (**h**) fourth derivative.

Secondly, calculations of wavelet transform were performed for each extrapolated vector. Finally, the expansion of the data was eliminated by cutting the results to the original size. As an initial step, the numerical results collected using a fine mesh (2 mm grid) were analyzed. The calculations of wavelet transforms were conducted using Gaussian wavelets with one to four vanishing moments. The fine scale *s* = 2 was initially set. To be compared with CWT, the conventional derivatives of the corresponding orders were determined. The comparison of both approaches is shown in Figure 6, where the damage maps are presented together with their cross-sections in the central part. Damage areas were marked on the charts. It is essential to note that the agreement between CWTs and derivatives is visible. The maps were similar in both approaches, the cross-sections were also comparable in shape for all wavelets. However, the derivatives included a considerable noise component that made the quality of the maps poorer and disrupted the possibility of the exact location of the damage. This observation allowed emphasizing the advantage of the CWT; the CWT maps had better quality when compared to the conventional derivatives, thanks to the smoothing function that reduced the noise. Thus, the derivatives were not used for further investigation. It is important to note that wavelets with a higher number of vanishing moments are more practical in the determination of the exact shape and position of defects. The damaged areas were revealed by *gaus1* wavelet; however, the defects could be incorrectly detected in intact sample #1. Nevertheless, CWT values for intact beam #1 are significantly lower than for damaged ones. This difference is not observed due to the individual scaling of each map (mutual scale could disturb the legibility of the results). What is more, the variability of CWT values in the area of debonding could suggest that there were multiple defects. These effects were not present for Gaussian wavelet with four vanishing moments that emphasized only the edges of the defect and flattened the areas with the same quality of adhesion. For this reason, the *gaus4* wavelet could be stated as the most effective for damage detection.

**Figure 7:**
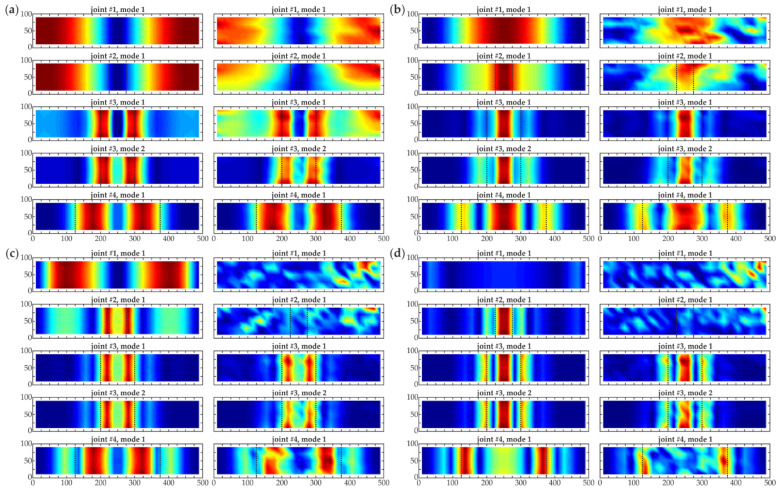
Comparison of CWT damage imaging for numerical (left column) and experimental (right column) results (grid 20 mm, no interpolation) for all joints #1–#4 (dimensions in [mm], scale *s* = 2) using different wavelets: (**a**) *gaus1*; (**b**) *gaus2*; (**c**) *gaus3*; (**d**) *gaus4*.

The second part of the analysis was the comparison between the numerical and experimental data. The results were obtained for a grid of 20 mm chosen based on the original mesh in experimental measurements (no additional interpolation was applied). As before, Gaussian wavelets were used with the constant scale *s* = 2. The CWT maps are shown in Figure 7. A good agreement between experimental and numerical maps was evident. Most of the maps were similar. However, the experimental results were demonstrably affected by the signal noise that deteriorated the quality of the obtained visualizations. As the degree of the wavelet increased, the influence of the noise became more visible, highlighting the differences between the experimental and numerical results, mainly for beams #1, #2 (the inconsistency between the maps obtained using *gaus3* and *gaus4* wavelets was clear). This effect made the localization of the debonding difficult, especially for the smallest damage (i.e., 5 cm (beam #2)), for which there was no possibility to detect the defect. As stated in the previous paragraph, the fourth order Gauss wavelet was the most powerful in the damage imaging. However, the quality of the maps was lower than those presented in Figure 6 due to the application of a coarser grid.

**Figure 8:**
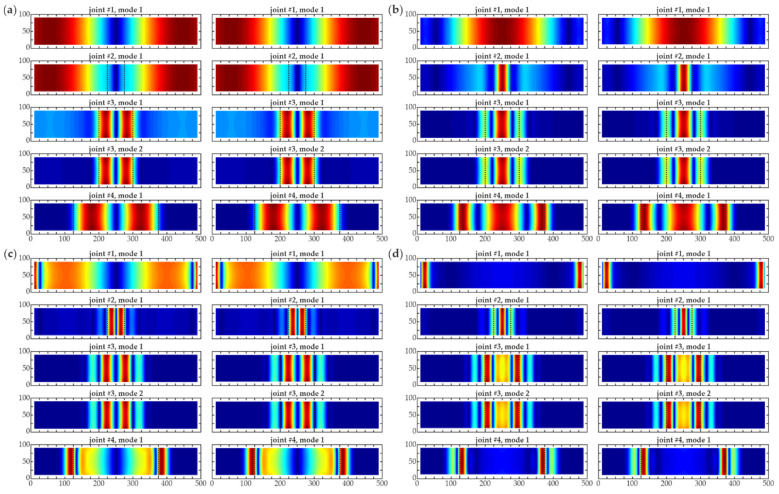
Comparison of CWT damage imaging for numerical results (left column—mesh 2 mm, no additional interpolation and right column—grid 20 mm interpolated to 2 mm, both for scale *s* = 8) for all joints #1–#4 (dimensions in [mm]) using different wavelets: (**a**) *gaus1*; (**b**) *gaus2*; (**c**) *gaus3*; (**d**) *gaus4*.

**Figure 9:**
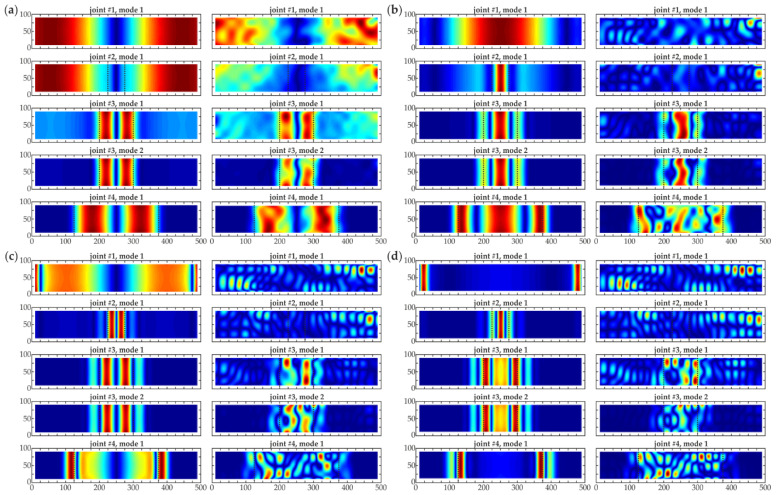
Comparison of CWT damage imaging for numerical (left column) and experimental (right column) results (grid 20 mm interpolated to 2 mm) for all joints #1–#4 (dimensions in [mm], scale *s* = 8) using different wavelets: (**a**) *gaus1*; (**b**) *gaus2*; (**c**) *gaus3*; (**d**) *gaus4*.

Additional interpolation was applied to the data measured on a 20 mm grid as an attempt to enhance the quality of the obtained maps. Firstly, the propriety of the proposed interpolation was verified based on the numerical results. The spline interpolation with the destined step of 2 mm was performed in MATLAB^®^. Taking into account the decrease in the step value, the scale for CWT calculations was increased to *s* = 8. Figure 8 presents the comparison of CWT maps obtained from originally fine mesh (2 mm) and interpolated from 20 to 2 mm. The accordance is clearly visible, proving the correctness of the performed interpolation. Furthermore, the numerical and experimental maps obtained using the above described interpolation are presented in Figure 9. It is firstly important to note that interpolation improved the quality of damage imaging for numerical data. The maps showed the debonding regions more clearly, the images were sharper, especially for the *gaus4* wavelet (Figure 9d). On the other hand, despite making the images sharper, the interpolation made the defect shape in the experimental maps more irregular, which may have been caused by the noise in the measured signals. The effect was much more visible for the higher-order wavelets (*gaus3*, *gaus4*), for which the boundaries of debonding regions became illegible. This was because high-order wavelets revealed small but sharp disturbances, which for experimental data could be both damage boundaries and noise, affecting the signals similarly. Based on this observation, it can be stated that the interpolation of noisy data collected in a coarse grid can enhance the quality of CWT damage visualization; however, high-order wavelets were not useful in this situation.

**Figure 10:**
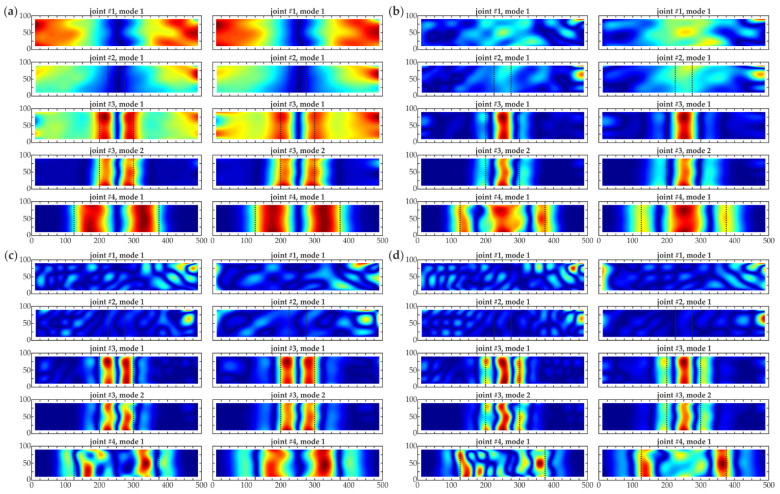
CWT damage imaging for experimental results (grid 20 mm interpolated to 2 mm) for all joints #1–#4 (dimensions in [mm]) using different wavelets for scales *s* = 16 (left column) and *s* = 24 (right column): (**a**) *gaus1*; (**b**) *gaus2*; (**c**) *gaus3*; (**d**) *gaus4*.

However, it was possible to change the scale to better visualize the damage in the experimental data. Figure 10 shows the CWT damage maps on the interpolated mesh (resulting grid of 2 mm) for multiplied scales, being doubling and tripling of the initial scale, i.e., *s* = 16 and *s* = 24. The positive influence of the increasing scale was evident. The shape of the debonding areas was clearly visible for both improved scales when compared to the initial value (cf. Figure 9), especially for high order wavelets. Larger scales highlighted the defects and allowed precise damage localization since they neglected the subtle signal noise. Some irregularities resulting from the noisy character of the experimental signals were visible. Comparing both increased scales, better results were obtained for the higher scale *s* = 24. However, the smallest defect (debonding of 5 cm) was still not detected.

## 4. Conclusions

The paper describes the non-destructive testing of concrete beams strengthened with steel plates. The issue of damage imaging in adhesive joints was considered. The modal analysis supported with the continuous wavelet transform was successfully applied. Gaussian wavelet family was assumed for calculations. Based on the obtained experimental and numerical results, the following conclusions could be formulated.

- The consistency between experimental and numerical eigenfrequencies and mode shapes confirmed the propriety of the performed experimental measurements and numerical simulations. The decrease of the natural frequency with the increasing size of the damaged area was observed.
- The interpretation of experimental and numerical mode shapes for all analyzed beams allowed initial damage detection by revealing significant disturbances connected with the presence of debonding areas.
- The comparison between conventional derivatives and continuous wavelet transforms for numerical results revealed the advantages of the latter. Both approaches gave consistent information about the damage; however, the CWT maps were more useful because of showing the defects more precisely.
- The appropriate choice of CWT calculation parameters is essential for the efficiency of obtained damage visualization. The quality of damage maps increased with the number of vanishing moments of the applied Gaussian wavelets. Low order of wavelet could lead to incorrect detection of defects in intact beams. In the case of scales, too high values could result in indistinct damage imaging, on the other hand, too low ones could reveal noise of signals.
- A good agreement between experimental and numerical CWT maps was observed for the data collected with coarse mesh (with a grid of 20 mm). However, the determination of the exact size of the smallest defect was not possible. The interpolation of the data allowed enhancing the quality of the obtained numerical damage maps. On the other hand, the experimental results had a poorer quality, because of the noise contained in the measured signals. The increase in scale helped overcome this difficulty.
- In general, the interpolation of the collected data can allow reducing the number of measurements. However, the coarse mesh grid can make the small defects undetectable. Furthermore, interpolation of experimental results can lead to the distortion of damage shape in CWT maps, especially for higher-order wavelets.

The final conclusion can be made that it is possible to determine the exact shape and position of the debonding in the adhesive joints of composite beams using modal analysis and continuous wavelet transform. However, the measurement mesh and CWT calculation parameters are important factors affecting the quality of the results. The proposed method is expected to have potential applications in the civil engineering industry. Having significantly greater sizes than laboratory specimens, real-scale engineering structures would require a division into smaller sections that could be analyzed in the way proposed in the current paper. This practical aspect of the paper could be the subject of future work. Another interesting direction for continuing the current research is the visualization of internal defects with smaller size and different shapes, such as the application of 2D wavelet transform.

## Figures and Tables

**Table 1:** Material parameters of the consisting elements of the composite beam.

Element	Material	Density *ρ* (kg/m^3^)	Elastic Modulus *E* (GPa)	Poisson’s Ratio *ν* (-)
Beam	Concrete	2364.4	48.0	0.16
Plate	Steel	7579.0	200.3	0.30
Film	Adhesive	1611.8	12.5	0.30

**Table 2:** Natural frequencies obtained numerically and experimentally for samples #1–4.

Sample	Mode	*f_num_* (Hz)	*f_exp_* (Hz)	Δ*f* (%)	*MAC* * (-)
#1	1	1899	1761	7.8	0.9991
#2	1	1898	1751	8.4	0.9892
#3	1	1859	1722	8.0	0.9899
	2	2468	2436	1.3	0.9782
#4	1	453	476	4.8	0.9966

* modal assurance criterion.

## Data Availability

The data underlying this article will be shared on reasonable request from the corresponding author.
